# Mobile Ecological Momentary Assessment and Intervention and Health Behavior Change Among Adults in Rakai, Uganda: Pilot Randomized Controlled Trial

**DOI:** 10.2196/22693

**Published:** 2021-07-20

**Authors:** Laura K Beres, Ismail Mbabali, Aggrey Anok, Charles Katabalwa, Jeremiah Mulamba, Alvin G Thomas, Eva Bugos, Gertrude Nakigozi, Mary K Grabowski, Larry W Chang

**Affiliations:** 1 Department of International Health Johns Hopkins Bloomberg School of Public Health Baltimore, MD United States; 2 Rakai Health Sciences Program Entebbe Uganda; 3 Department of Epidemiology University of North Carolina, Chapel Hill Chapel Hill, NC United States; 4 Department of Surgery Johns Hopkins University Baltimore, MD United States; 5 University of Chicago Pritzker School of Medicine Chicago, IL United States; 6 Department of Population, Family and Reproductive Health Johns Hopkins Bloomberg School of Public Health Baltimore, MD United States; 7 Department of Internal Medicine University of Pittsburgh Medical Center Pittsburgh, PA United States; 8 Department of Pathology Johns Hopkins University School of Medicine Baltimore, MD United States; 9 Division of Infectious Diseases Department of Medicine Johns Hopkins University School of Medicine Baltimore, MD United States

**Keywords:** ecological momentary assessment, ecological momentary intervention, mHealth, digital health, smartphone, mobile phone, randomized trial, Uganda, Africa

## Abstract

**Background:**

An extraordinary increase in mobile phone ownership has revolutionized the opportunities to use mobile health approaches in lower- and middle-income countries (LMICs). Ecological momentary assessment and intervention (EMAI) uses mobile technology to gather data and deliver timely, personalized behavior change interventions in an individual’s natural setting. To our knowledge, there have been no previous trials of EMAI in sub-Saharan Africa.

**Objective:**

To advance the evidence base for mobile health (mHealth) interventions in LMICs, we conduct a pilot randomized trial to assess the feasibility of EMAI and establish estimates of the potential effect of EMAI on a range of health-related behaviors in Rakai, Uganda.

**Methods:**

This prospective, parallel-group, randomized pilot trial compared health behaviors between adult participants submitting ecological momentary assessment (EMA) data and receiving behaviorally responsive interventional health messaging (EMAI) with those submitting EMA data alone. Using a fully automated mobile phone app, participants submitted daily reports on 5 different health behaviors (fruit consumption, vegetable consumption, alcohol intake, cigarette smoking, and condomless sex with a non–long-term partner) during a 30-day period before randomization (P1). Participants were then block randomized to the control arm, continuing EMA reporting through exit, or the intervention arm, EMA reporting and behavioral health messaging receipt. Participants exited after 90 days of follow-up, divided into study periods 2 (P2: randomization + 29 days) and 3 (P3: 30 days postrandomization to exit). We used descriptive statistics to assess the feasibility of EMAI through the completeness of data and differences in reported behaviors between periods and study arms.

**Results:**

The study included 48 participants (24 per arm; 23/48, 48% women; median age 31 years). EMA data collection was feasible, with 85.5% (3777/4418) of the combined days reporting behavioral data. There was a decrease in the mean proportion of days when alcohol was consumed in both arms over time (control: P1, 9.6% of days to P2, 4.3% of days; intervention: P1, 7.2% of days to P3, 2.4% of days). Decreases in sex with a non–long-term partner without a condom were also reported in both arms (P1 to P3 control: 1.9% of days to 1% of days; intervention: 6.6% of days to 1.3% of days). An increase in vegetable consumption was found in the intervention (vegetable: 65.6% of days to 76.6% of days) but not in the control arm. Between arms, there was a significant difference in the change in reported vegetable consumption between P1 and P3 (control: 8% decrease in the mean proportion of days vegetables consumed; intervention: 11.1% increase; *P*=.01).

**Conclusions:**

Preliminary estimates suggest that EMAI may be a promising strategy for promoting behavior change across a range of behaviors. Larger trials examining the effectiveness of EMAI in LMICs are warranted.

**Trial Registration:**

ClinicalTrials.gov NCT04375423; https://www.clinicaltrials.gov/ct2/show/NCT04375423

## Introduction

### Background

To date, behavior change strategies in lower- and middle-income countries (LMICs) have failed to fully leverage the potential of mobile technology to promote optimal health outcomes. Although this may be partially because of historically limited technology access in these settings, an extraordinary increase in mobile technology ownership and use, facilitated by advances in lower-cost smartphones, has revolutionized the opportunities to use mobile health approaches in LMICs [1].

Ecological momentary assessment and intervention (EMAI) uses mobile technology to gather individual-level behavioral data and deliver timely, personalized behavior change interventions in an individual’s natural setting [2]. These strengths can promote a range of health objectives. Compared with traditional, in-person assessments and interventions, EMAI may offer more user-driven, cost-effective, and ecologically and temporally relevant strategies [2,3] and may generate more accurate data than traditional retrospective questionnaires, which are subject to recall bias [4]. Rapid and repeated individual-level behavioral measurement and feedback may be particularly effective in supporting changes to semiconscious behaviors or habits that are difficult to accurately recall and benefit from interruptions in routine to alter [5,6]. Remote data collection and intervention strategies may be critical for hard-to-reach populations, and because of infection-related concerns such as COVID-19, this may help to fill the more widespread need for remote or contactless intervention options.

However, there is limited extant literature on the effectiveness of EMAI, particularly in LMICs. Several studies in high-income settings have demonstrated the preliminary effectiveness of EMAI using targeted, remote messages to improve mental health outcomes [3,7,8], fruit and vegetable consumption [9], and smoking-related behaviors [10]. A qualitative study in the United States demonstrated that young women responded positively to the development of an EMAI approach for sexual risk reduction, identifying the potential for future intervention trials [11]. There are several recently published protocols on the use of mobile ecological momentary intervention across behaviors, including alcohol and drug use, healthy food consumption and coping [12-16], and a consistently identified need for more research into the effectiveness of EMAI with mobile technologies [17-19]. To date, we are not aware of any previous EMAI trials in sub-Saharan Africa.

### Objectives

To advance the evidence base for mobile health interventions in LMICs, we conducted a pilot randomized trial to establish estimates of the potential effect of EMAI on a broad range of health-related behaviors in Rakai, Uganda. On the basis of extant EMAI literature, theory, and evidence [20] that targeted nudges, including behavioral messaging, can alter behavior, we hypothesized that participants submitting ecological momentary assessment (EMA) reports and receiving intervention messaging (EMAI) would have improved self-reported health behaviors compared with those submitting EMA reports only. As the first study, to our knowledge, to trial EMAI in sub-Saharan Africa, we sought to generate preliminary data to guide future investigations on the feasibility and effectiveness of EMAI in LMICs.

## Methods

### Study Design and Population

The study was a prospective, parallel-group, randomized pilot trial in Rakai, Uganda. It sought to establish a preliminary estimate of the effect of EMAI on health behaviors between participants submitting EMA data and receiving behaviorally responsive interventional health messaging compared with those submitting EMA data alone.

The study sampled adult participants (aged 18-49 years) from the Rakai Community Cohort Study (RCCS), an open, population-based cohort running since 1994 [21,22]. Rakai District, Uganda, is approximately 150 km southwest of the capital, Kampala, bordered by Tanzania and Lake Victoria. It includes agrarian, trading, and fishing communities [22]. Participants were eligible if they were current RCCS participants who had provided a telephone number during the last survey and had at least a secondary-level education. The lists of potential participants who met the eligibility criteria according to the RCCS survey data were generated from the RCCS database. Participants were purposively recruited via the telephone. Study staff members sought variation in participant age, sex, and occupation, aiming to include a minimum of 20% traders and 20% farmers in the sample, to enable researchers to assess possible differential EMAI acceptability and feasibility by participant characteristics in this pilot trial.

In addition to the primary study outcome, the preliminary estimate of the effect of EMAI, as a pilot study, the secondary aim was to assess the feasibility of the data collection and intervention approach. To do so, we examined the indicators of data collection success by the study arm. All outcomes were assessed after the closure of the study.

### Procedures

Interested participants attended an in-person visit at the study office, enrolling on completion of voluntary, written informed consent at the first in-person study visit. At the first study visit, participants were issued a password-protected smartphone programmed with the EMAI study app (emocha Health Inc), a phone charger, and a portable power bank. Participants were trained on the use of the smartphone and a fully automated study app and completed a paper-based enrollment questionnaire collecting participant demographic and behavioral data, recalling the 30 days before enrollment.

Communicating in Luganda, the primary language spoken in the region, the app collected EMA data on 5 behaviors of interest: fruit consumption, vegetable consumption, alcohol intake, cigarette smoking, and sex with a nonmarital or non–long-term partner without a condom. EMA behavioral data were submitted by the participant through the app (1) in response to a text message prompt twice per day, once at a random time and once at a fixed time asking about each of the behaviors since the last prompt-based report, (2) in response to a text message prompt sent each week, recalling behaviors throughout the week, and (3) through a participant-generated report sent within approximately 1 hour of engaging in any of the study behaviors of interest (an *event-contingent* report). Participants were asked to reply “yes=1” or “no=0” to sequential questions about each behavior. If they replied “yes,” they were asked for the associated numeric quantity (eg, number of cigarettes smoked and number of vegetables eaten).

**Figure 1:**
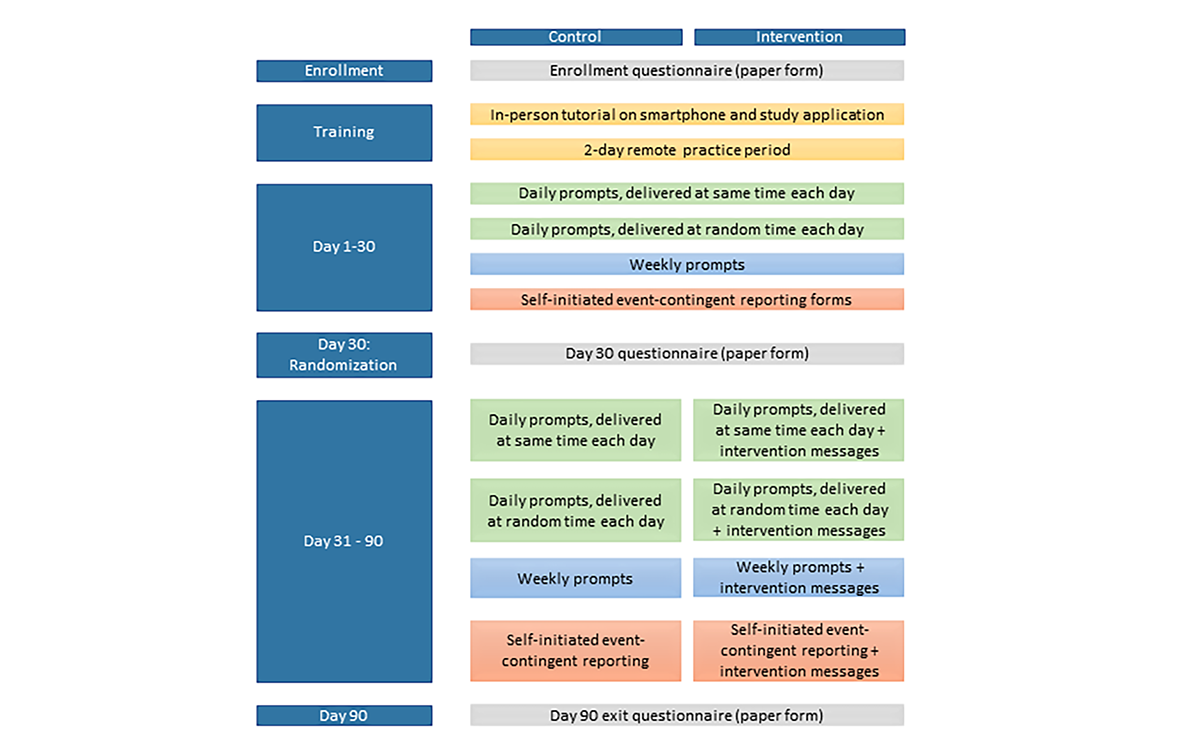
Study design.

For the first 30 days of the study, all participants sent EMA data, after which they were randomized to control or intervention conditions. Participants in the control arm continued to submit EMA data throughout the remainder of the study period. From randomization to study exit, intervention arm participants received health-related messages responsive to the behavioral data submitted in addition to continuing EMA data submissions. The messages, developed using participatory formative research including free-listing and sorting of proposed messages with a convenience sample of 8 Rakai residents to enhance appropriateness and relevance, provided positive reinforcement for reported healthy behaviors (eg, Living alcohol-free today is a step to a healthier future! Alcohol contributes to heart disease and liver cancer.) and encouragement to change in response to reported risk behaviors (eg, Alcohol abuse increases your risk of heart disease. Protect your heart, and stick to water or juice tomorrow). The participants received messages that were directly relevant to the responses they submitted. The specific message a participant received from the bank of possible messages (Multimedia Appendix 1) related to the reported behavior was randomly selected each time. Participants exited after study day 90 (Figure 1). Up to 10 participants were enrolled simultaneously throughout the study period. Behavioral data were stored on the phone and sent to the remote study database for analysis.

Participants were compensated for their time (UGX 10,000; approximately US $3) and reimbursed for travel costs (UGX 5000-40,000; US $1.50-12) for each in-person study visit. Participants were given funds equivalent to 525 MBs of data monthly throughout the study and an incentive totaling UGX 100,000 (approximately US $30) in 3 increments at 30, 60, and 90 days for responding to ≥50% of data collection prompts. The study was approved by the Ugandan Virus Research Institute Research and Ethics Committee and the Johns Hopkins School of Medicine Institutional Review Board.

### Randomization

Participants were assigned to the control or intervention study arm using block randomization with randomly varying block sizes of 4, 6, and 8 through blockr R package by Greg Snow. The study arm assignments were enclosed within opaque, consecutively numbered envelopes. At the day 30 visit, the study coordinator allocated the randomization assignment enclosed in the next consecutive envelope to each participant, activating the appropriate EMAI smartphone module. The assignments were not masked to the study participants or staff.

### Study Measurements and Outcomes

Participant characteristics and behaviors at enrollment were collected on the paper-based enrollment questionnaire. Occupation was measured using the last RCCS survey round. The exposure of interest, receiving intervention messages, was measured as a dichotomous variable, with all participants assigned to the intervention arm counted as exposed and all control arm participants as unexposed. The outcomes were examined separately for each of the 5 study behaviors of interest: (1) fruit consumption, (2) vegetable consumption, (3) alcohol use, (4) cigarette smoking, and (5) sex with a nonmarital or non–long-term partner without a condom.

Participants were assigned a *yes* or *no* for each behavior for each day of the study follow-up. A participant was counted as engaging in a behavior if the participant reported having practiced the behavior on at least one of the twice-daily prompt response forms or any event-contingent form submitted on that day. They were counted as not engaging in the behavior if none of the submitted forms reported the behavior on that day. If no data forms were submitted, the participant had missing data for that day. Sex with a nonmarital or non–long-term partner without a condom was determined by 2 questions: first, asking if the participant had a sexual encounter with such a partner and then asking if a condom was used in that encounter. Participants were counted as engaging in the behavior if they reported both *yes* to sex with a nonmarital or non–long-term partner and *no* to condom use in that encounter.

The total number of days in the study was counted from enrollment to the exit date. The number of event-contingent reports and prompt-driven behavioral report responses submitted was counted using the total number of database entries (submitted by the smartphone and received by the database) for each type of report mechanism. Each report included the behavioral information reported and the time and date it was submitted. A day was counted as missing behavioral data for a participant if there were no reports recorded in the database on a date between study enrollment and exit. The study follow-up was divided into 3 study periods: period 1 (baseline; P1: enrollment to the day before randomization), period 2 (P2: the day of randomization to 29 days postrandomization), and period 3 (P3: 30 days postrandomization to study exit).

### Analysis

Given the pilot nature of the study, we primarily used a descriptive approach to examine the study outcomes. Descriptive statistics were used to compare participant characteristics and data collection between the 2 study arms. We examined the comparability of participant characteristics by arm at baseline using chi-square and two-tailed Student *t* tests. We estimated the effect sizes for differences in data collection between the 2 arms using Cohen *d* with bootstrapped CIs to account for the small sample size and nonnormal data distribution. We determined the proportion of study days when a behavior was practiced by taking the total number of days the participant engaged in the behavior over the total days with behavioral data reported for each participant for each study period. We excluded missing data (days without behavioral reports). We calculated bootstrapped 95% CIs to identify differences in the mean proportion of days when participants reported each behavior between P1 and P2, P2 and P3, and P1 and P3 within each study arm. We present visual plots of the proportion of days when behaviors are reported for each participant by study arm and study period. To compare changes between study arms, we calculated the mean difference in the proportion of days each participant reported each behavior between periods by subtracting the later period’s mean proportion of days when the behavior was practiced from the earlier period’s mean. We then took the overall mean difference across all participants for each study arm and calculated the difference within the differences by subtracting the control arm from the intervention arm. We used two-sample *t* tests with two-sided *P* values to assess if the mean differences between each period were the same in the 2 study arms. The analyses were conducted using Stata 15.1 IC (StataCorp, 2018).

## Results

### Overview and Flow

**Figure 2:**
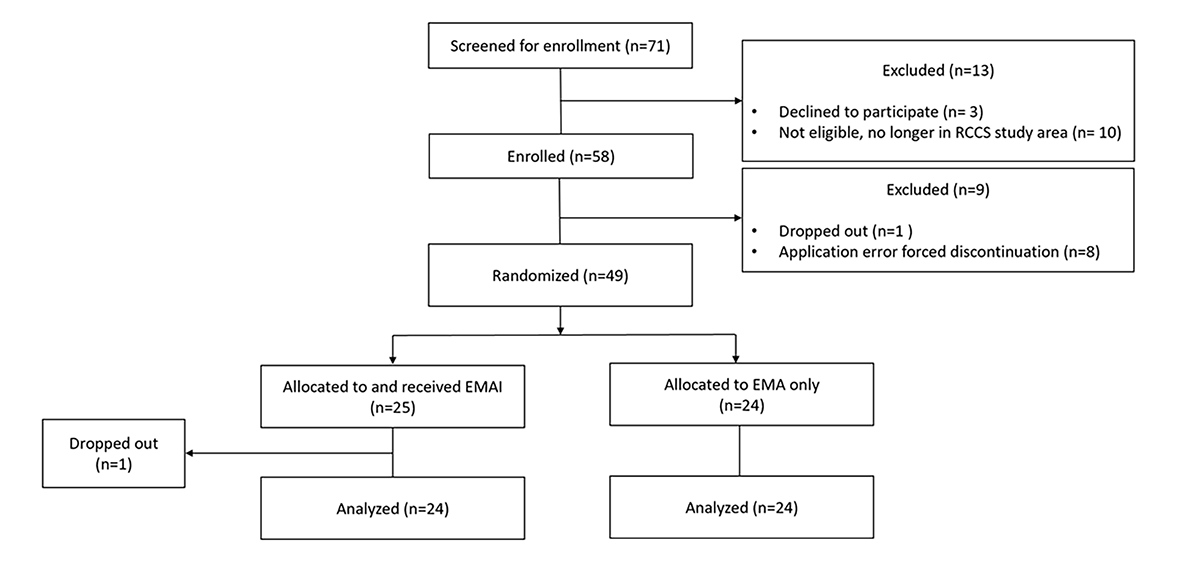
Participant flow diagram. EMA: ecological momentary assessment; EMAI: ecological momentary assessment and intervention.

Between June 10, 2016, and March 1, 2017, 71 participants were screened for enrollment, of whom 58 were enrolled. Of 58 participants, 8 were excluded because of early failure of the study application and 2 dropped out at 15 and 79 days after enrollment. The complete analysis data set included 48 participants (Figure 2).

Of the 48 participants, 23 (48%) were female, with a median age of 31 (IQR 25-38) years. Less than one-third of the participants worked in agriculture (14/48, 29%), with 17% (8/48) working in trade, 23% (11/48) teachers, and 31% (15/48) in other occupations. In the 30 days before study enrollment, nearly all participants (47/48, 98%) reported eating a vegetable on at least one day, 90% (43/48) consumed fruit, whereas 35% (17/48) consumed alcohol, 17% (8/48) reported sex with a nonmarital or non–long-term partner, and 13% (3/48) smoked a cigarette. There were no significant differences in participant characteristics or behaviors at enrollment between the study arms (Table 1).

**Table 1:** Participant characteristics at enrollment by study arm.

Participant characteristics			Control (n=24)		Intervention (n=24)		Total (N=48)		*P* value^a^	
Female, n (%)			12 (50)		11 (46)		23 (48)		.77	
Age at enrollment (years), mean (SD)			32.7 (7.1)		30.1 (6.7)		31.4 (7.0)		.10	
**Education completed** **, n (%)**										.94
	Some secondary	7 (29)		8 (33)		15 (31)				
	Secondary	10 (42)		9 (38)		19 (40)				
	University, technical or vocational	7 (29)		7 (29)		14 (29)				
Yes, owns a cell phone, n (%)			24 (100)		24 (100)		48 (100)		N/A^b^	
Yes, feels comfortable using a phone to send text messages, n (%)			24 (100)		22 (92)		46 (96)		.15	
Yes, ever used a smartphone app, n (%)			14 (58)		12 (50)		26 (54)		.56	
**Occupation** **, n (%)**										.53
	Agrarian	6 (25)		8 (33)		14 (29)				
	Trader	5 (20)		3 (12)		8 (16)				
	Teacher	4 (17)		7 (29)		11 (23)				
	Other	9 (38)		6 (25)		15 (31)				
**Health behaviors, past 30 days**										
	Smoked cigarette at least one day, n (%)	3 (13)		0 (0)		3 (13)		.07		
	Among smokers, days smoked at least one cigarette, mean (SD)	20 (13.1)		N/A		N/A		N/A		
	Drank alcoholic beverage at least one day, n (%)	8 (33)		9 (38)		17 (35)		.76		
	Among drinkers, days drank at least one alcoholic beverage, mean (SD)	2.1 (1.3)		1.6 (0.7)		N/A		.29		
	Ate vegetables at least one day, n (%)	22 (92)		21 (88)		43 (90)		.64		
	Among those who ate vegetables, days ate at least one vegetable, mean (SD)	7.2 (5.4)		6.7 (7.9)		N/A		.80		
	Ate fruit at least one day, n (%)	23 (96)		24 (100)		47 (98)		.31		
	Among those who ate fruit, days ate at least one fruit, mean (SD)	13.6 (9.0)		12.5 (8.2)		N/A		.67		
	Had sex with nonmarital or non–long-term partner without using a condom at least once, n (%)	5 (21)		3 (13)		8 (17)		.44		
	Times had sex with a nonmarital or non–long-term partner without a condom, among those reporting sex, mean (SD)	2.4 (1.9)		2.3 (0.6)		N/A		.96		

^a^Two-sided *P* value calculated using chi-square tests for categorical variables and Student *t* test for continuous variables.

^b^N/A: not applicable.

### Data Collection

The mean total number of days of follow-up was 92 (minimum 90 and maximum 94). Comparing study arms, there were no significant differences in time in study, data submission types (event-contingent or prompt-based responses), or proportion of study days without data submitted (Table 2). There were also no significant differences in study arm in behaviors reported during the prerandomization P1 baseline period (Table 2). Overall, 85.5% (3777/4418) of the total study days had behavioral data reported.

Over the study periods, the reported engagement in any of the behaviors varied. All 48 participants reported eating fruits and vegetables on at least one day during each of the 3 study periods, except for 1 participant who did not report eating vegetables on any day during study period 2. Ever consuming alcohol was reported by approximately half of the participants or fewer across the periods (control arm period 1: 13 participants, period 2: 9 participants, and period 3: 8 participants; intervention arm periods 1 and 2: 12 participants, period 3: 7 participants). Far fewer participants reported ever having sex with a non–long-term partner without a condom (control arm period 1: 4 participants, periods 2 and 3: 2 participants; intervention arm periods 1 and 2: 4 participants and period 3: 5 participants) or ever smoking cigarettes (control arm period 1: 7 participants, period 2: 3 participants, and period 3: 4 participants; intervention arm period 1: 3 participants, period 2: 1 participant, and period 3: 0 participants).

**Table 2:** Study data collection indicators by study arm.

Data indicator			Control		Intervention		*t* test^a^ (*df*)		*P* value^a^		Effect size^b^ (95% CI)
Total study days, mean (range)			92.0 (90-94)		92.1 (90-94)		−0.28 (46)		.78		−0.08 (−0.66 to 0.50)
**Days in study period, mean (range)**											
	Period 1: baseline (study day 1 to day before randomization)	30.6 (29-33)		30.8 (29-33)		−0.57 (46)		.57		−0.16 (−0.71 to 0.38)	
	Period 2 (randomization to 29 days postrandomization)	30 (30-30)		30 (30-30)		N/A^c^		N/A		N/A	
	Period 3 (30 days postrandomization to final study day)	31.4 (28-33)		31.3 (28-33)		0.23 (46)		.82		0.07 (−0.48 to 0.61)	
Total event-contingent reports, mean (SD)			108.6 (67.5)		99.8 (46.4)		0.53 (46)		.60		0.15 (−0.44 to 0.75)
**Event-contingent reports by period, mean (SD)**											
	Period 1: baseline (study day 1 to day before randomization)	47.5 (35.0)		43.3 (26.7)		0.47 (46)		.63		0.14 (−0.45 to 0.72)	
	Period 2 (randomization to 30 days after randomization)	30.0 (22.4)		27.9 (14.7)		0.38 (46)		.71		0.11 (−0.54 to 0.76)	
	Period 3 (30 days after randomization to final study day)	31.1 (23.0)		28.9 (13.8)		0.46 (46)		.65		0.13 (−0.48 to 0.75)	
Total responses submitted to prompts, mean (SD)			92.2 (28.6)		96.9 (23.2)		−0.63 (46)		.53		−0.18 (−0.78 to 0.42)
**Responses submitted to prompts by period, mean (SD)**											
	Period 1: baseline (study day 1 to day before randomization)	20.4 (6.9)		21.5 (5.0)		−0.62 (46)		.54		−0.18 (−0.78 to 0.42)	
	Period 2 (randomization to 30 days after randomization)	35.4 (14.5)		38.3 (9.0)		−0.85 (46)		.40		−0.24 (−0.82 to 0.33)	
	Period 3 (30 days after randomization to final study day)	36.4 (12.8)		37.1 (12.2)		−0.18 (46)		.85		−0.05 (−0.64 to 0.53)	
Total days without behavior reported, mean (SD)			14.2 (10.7)		12.4 (9.3)		0.62 (46)		.54		0.18 (−0.45 to 0.81)
**Days without behavior reported by period, mean (SD)**											
	Period 1 (study day 1 to day before randomization)	3.1 (2.6)		3.4 (2.9)		−0.36 (46)		.72		−0.10 (−0.70 to 0.49)	
	Period 2 (randomization to 30 days after randomization)	3.7 (3.2)		3.0 (3.1)		0.73 (46)		.47		0.21 (−0.44 to 0.86)	
	Period 3 (30 days after randomization to final study day)	7.4 (7.1)		6.0 (6.2)		0.74 (46)		.47		0.21 (−0.40 to 0.82)	
Proportion of study days without behavior report (%), mean (SD)			15.4 (11.5)		13.5 (10.1)		0.61 (46)		.54		0.18 (−0.45 to 0.81)
**Proportion of study days participants report behaviors in first study period: P1 baseline (prerandomization; %), mean (SD)**											
	Fruit	79.0 (0.2)		78.6 (0.2)		0.06 (46)		.95		0.02 (−0.61 to 0.65)	
	Vegetable	57.8 (0.2)		65.6 (0.3)		−0.99 (46)		.33		−0.28 (−0.93 to 0.36)	
	Alcohol	9.6 (0.2)		7.2 (0.1)		0.51 (46)		.61		0.15 (−0.44 to 0.73)	
	Sex with non–long-term partner without a condom	1.9 (0.04)		6.6 (0.1)		−1.74 (46)		.09		−0.50 (−0.96 to −0.05)	
	Smoking	6.5 (0.2)		1.4 (0.03)		1.26 (46)		.22		0.36 (−0.14 to 0.86)	

^a^Two-sided *P* value calculated using Student *t* test for continuous variables.

^b^Cohen *d*.

^c^N/A: not applicable.

### Within-Arm Change Over Time

**Figure 3:**
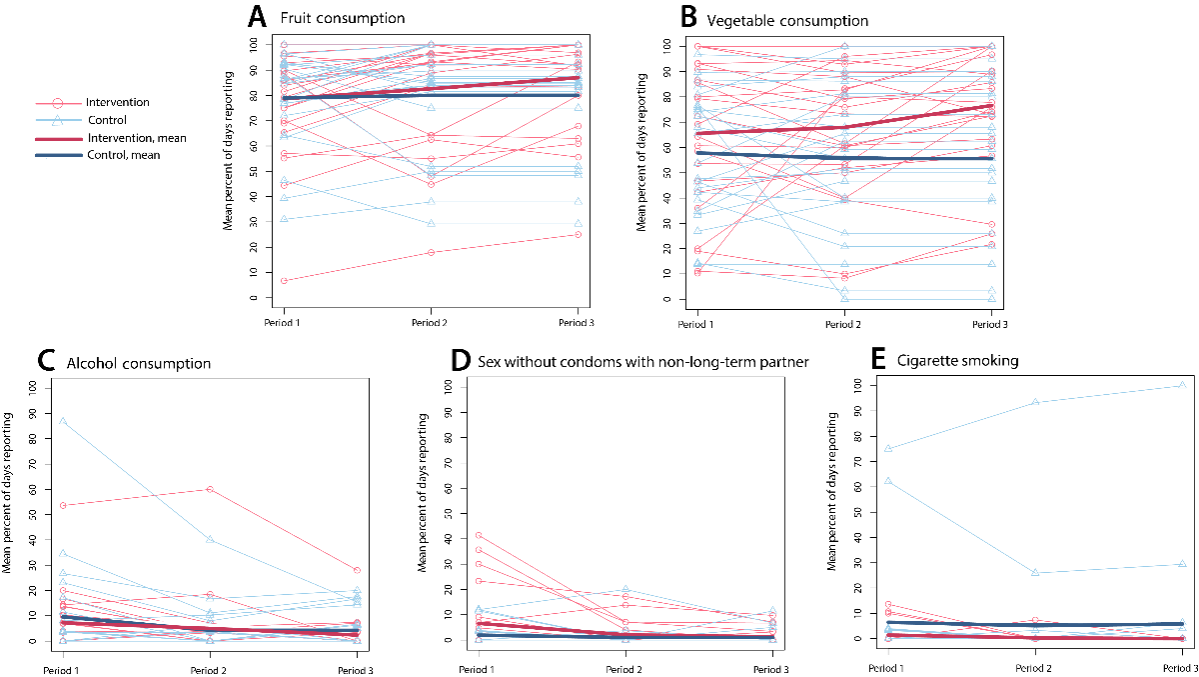
Mean proportion of days on which behavior occurred by participant according to study arm and period (n=24 per arm).

There was a decrease in the mean proportion of days when alcohol was consumed in both the control and intervention arms. In the control arm, a decrease was observed between periods 1 and 2 (9.6% of days to 4.3% of days), whereas it was observed between periods 1 and 3 in the intervention arm (7.2% of days to 2.4% of days; Table 3). Similarly, both arms showed a decrease in the mean proportion of days when participants reported having sex with a nonmarital or non–long-term partner without a condom between periods 1 and 3 (control: 1.9% of days to 1% of days and intervention: 6.6% of days to 1.3% of days) and an increase in fruit consumption (control: 79% of days to 82% of days and intervention: 78.6% of days to 87% of days; Table 3). In the intervention arm only, there was an increase in the mean proportion of days when vegetables were reported to be consumed between periods 1 and 3 (vegetable: 65.6% of days to 76.6% of days) and periods 2 and 3 (vegetable: 68% of days to 76.6% of days; Table 3; Figure 3).

**Table 3:** Mean proportion of days participants report behaviors by study period and arm (n=24 per arm).

Reported behavior	Control (%), mean (95% CI)				Intervention (%), mean (95% CI)		
	Period 1	Period 2	Period 3	Period 1		Period 2	Period 3
Fruit	79 (71.9 to 86)	80 (71.3 to 88.8)	82 (73.7 to 90.3)	78.6 (70.2 to 87.1)		82.6 (73.5 to 91.8)	87.0 (79.3 to 94.7)
Vegetable	57.8 (48.2 to 67.5)	55.6 (44.2 to 66.9)	49.9 (37.7 to 62)	65.6 (54.1 to 77)		68 (57.3 to 78.6)	76.6 (67 to 86.2)
Alcohol	9.6 (1.3 to 17.8)	4.3 (1 to 7.6)	4.2 (1.6 to 6.8)	7.2 (2.9 to 11.6)		5 (−0.1 to 10.1)	2.4 (−0.2 to 4.9)
Sex with non–long-term partner without a condom	1.9 (0.5 to 3.3)	1 (−0.7 to 2.7)	1 (−0.1 to 2.1)	6.6 (1.8 to 11.4)		2.1 (0.3 to 3.8)	1.3 (0.2 to 2.3)
Smoking	6.5 (−0.9 to 13.9)	5.1 (−2.6 to 12.8)	5.8 (−2.7 to 14.3)	1.4 (−0.1 to 3)		0.3 (−0.3 to 1)	0^a^

^a^95% CI values are not applicable.

### Between-Arm Comparison

The comparison of study arms showed a significant difference in the change in reported vegetable consumption between periods 1 and 3 (control: 8% decrease in the mean proportion of days vegetables were consumed; intervention: 11.1% increase in the mean proportion of days vegetables were consumed; *P*=.01) and periods 2 and 3 (control: 5.7% decrease in the mean proportion of days vegetables were consumed; intervention: 8.6% increase in the mean proportion of days vegetables were consumed; *P*=.002). There were no other significant differences in the change in behavior over time between the intervention and control groups (Table 4).

**Table 4:** Mean difference in proportion of days participants reported behavior between periods (later period minus earlier period, positive number indicates increase in behavior over time, and negative number indicates decrease in behavior over time; n=24 per arm).

Reported behavior		Control (%)	Intervention (%)	Difference of differences (intervention−control; %)	*P* value^a^
**Period 1 to period 2**					
	Fruit	1.01	3.99	2.98	.49
	Vegetable	−2.28	2.41	4.69	.47
	Alcohol	−5.28	−2.24	3.04	.24
	Sex with non–long-term partner without a condom	−0.94	−4.53	−3.59	.12
	Smoking	−1.36	−1.12	0.24	.90
**Period 2 to period 3**					
	Fruit	1.98	4.38	2.4	.52
	Vegetable	−5.71	8.64	14.35	.002
	Alcohol	−0.05	−2.61	−2.56	.21
	Sex with non–long-term partner without a condom	−0.02	−0.8	−0.78	.42
	Smoking	0.71	−0.31	−1.02	.07
**Period 1 to period 3**					
	Fruit	2.99	8.37	5.38	.18
	Vegetable	−7.99	11.05	19.04	.01
	Alcohol	−5.33	−4.86	0.47	.89
	Sex with non–long-term partner without a condom	−0.96	−5.33	−4.37	.07
	Smoking	−0.65	−1.43	−0.78	.69

^a^Two-sided *P* values calculated using two-sample *t* test.

## Discussion

### Principal Findings

This pilot study found that EMAI was feasible and may influence a range of participant behaviors. EMA alone may also affect the reported behaviors. To our knowledge, this is the first study of EMAI in sub-Saharan Africa. It provides a foundation on which further research on EMAI in transferable settings can be framed.

The feasibility of EMAI in a pilot study context in Rakai, Uganda, was supported in this study through high participant retention in both arms, yielding comparable study groups without adjustment and consistent submission of EMA data. The 85.5% (3777/4418) of the combined study follow-up days with behavioral data in this study is consistent with or better than data collection feasibility in other studies in high-income settings [3,23-26]. Compared with study periods 1 and 2, study period 3 saw an increase in days without behavioral reports in both study arms, indicating that more support may be needed to maintain EMA reports over time. Other EMAI studies have discussed the need for a careful examination of the participant population and study procedure burden to determine necessary and appropriate support for successful EMAI implementation [26,27].

Remote data collection and messaging may have some effect on behavior over a relatively short period. Descriptive comparisons of daily behavioral reports between the approximately 30-day study periods within arms suggest that alcohol consumption, sex with a non–long-term partner without a condom, fruit consumption, and smoking may be influenced by EMA or EMAI and that vegetable consumption may be influenced by EMAI. Although the study expected behavior change to be associated with intervention messaging, we hypothesize that daily reporting increased participant awareness of health risk behaviors, which may have changed their practices or reporting. This is consistent with theoretical and interventional extant self-monitoring literature, supporting that reactivity associated with improved self-awareness, particularly for routinized behaviors, can lead to behavior change [28,29].

Future research should not only expand beyond a pilot context to determine more robust estimates of the EMAI effect but should also examine differential pathways of change in raising awareness of risk behaviors compared with provision of feedback on positive, routinized behaviors to better understand the potential of both EMA and EMAI, beyond measurement. Although behavioral change may be rapid with EMAI, it is also necessary to examine the sustainability of change beyond the study’s relatively short 90-day follow-up. Although our study used smartphones to allow for geospatial data collection in addition to behavioral data exchange, any phone with SMS or Unstructured Supplementary Service Data capabilities could support the key elements of the study monitoring and intervention. Research examining the effect of EMAI using more basic phones could broaden the reach of future EMAI work by allowing interventions to operate on any type of phone currently owned by members of the population of interest.

Throughout the study period, the direction of the intervention arm trends in the mean proportion of days when behaviors were reported was consistent: increasing for fruit and vegetable consumption and decreasing for alcohol consumption, cigarette smoking, and sex with a nonmarital or non–long-term partner without a condom. There was more variation in the trends in the control arm. Although only vegetable consumption showed significant differences in change over time between arms, the direction and, with the exception of alcohol, the magnitude of the change in behaviors between study arms are consistent with the study hypotheses. This supports that remote intervention messaging warrants further study to promote behavioral change. Remote data collection and intervention may be particularly important in LMIC contexts such as Rakia, Uganda, where regular follow-up of people is difficult because of high population mobility and poor infrastructure. Similarly, in the era of COVID-19, human interaction carries risks that may exceed small to moderate, but otherwise important, behavior change benefits. Research to further establish the effectiveness of EMAI in these settings may be of critical importance.

### Limitations

The findings of this pilot trial were not designed to be generalizable beyond the study’s target population, including participants with at least a secondary level of education or a 90-day follow-up period. However, as preliminary estimates, they offer insight into the potential of EMAI and warrant further exploration. Behaviors were self-reported. It is not possible to differentiate actual changes in behavior from changes in reported behaviors influenced by social desirability bias, potentially reinforced by intervention messages, or other facts. However, although at different magnitudes, changes were observed in both the control and intervention arms of the trial. Furthermore, for sensitive behaviors such as condom use, self-report is the best available standard, with questions asking about recent experiences considered to be more valid and reliable than longer recall periods [30]. We did not collect the servings of fruits and vegetables consumed, precluding our ability to examine a dose-response relationship, which would be of interest in future, larger trials.

The study enrolled current RCCS participants who are accustomed to participating in trials. They may respond differently to interventions than research-naïve participants. Given the pilot nature of the study, explanations for reported behavioral changes other than the effect of intervention messaging cannot be ruled out. Particularly given block randomization and the study limit of 10 simultaneous active participants, seasonality in vegetable access, for example, could have influenced the decrease observed in vegetable consumption in the control arm.

### Conclusions

Preliminary estimates from this pilot trial suggest that EMAI may be an effective strategy to promote behavior change across a range of behaviors. Larger trials examining the effectiveness of EMA alone and EMAI with responsive messaging in LMICs are warranted. Cost-effectiveness work is also recommended to establish the comparative potential of EMAI with more traditional approaches, leveraging increasingly accessible mobile technology in low-resource settings.

## Appendix

Multimedia Appendix 1 Bank of possible messages sent in response to reported behaviors (English translations).

Multimedia Appendix 2 CONSORT-eHEALTH checklist V1.6.1.

## Abbreviations

EMA: ecological momentary assessment
EMAI: ecological momentary assessment and intervention
LMIC: lower- and middle-income country
RCCS: Rakai Community Cohort Study
