# Supplementary material for: Mobile Ecological Momentary Assessment and Intervention and Health Behavior Change Among Adults in Rakai, Uganda: Pilot Randomized Controlled Trial
Source: JMIR Form Res. 2021 Jul 20;5(7):e22693. doi: 10.2196/22693 (PMC8335611; doi:10.2196/22693)
Supplement: Multimedia Appendix 1 [file formative_v5i7e22693_app1.docx]

**Multimedia Appendix**

**Table 1. Bank of possible messages sent in response to reported behaviors (English translations)**

| **Supportive of not smoking** |
| --- |
| You've set a great example for your loved ones by not smoking today. Great job! |
| Living a smoke-free life is an investment in your future. Good job! |
| That's fantastic. Instead of investing in cigarettes, you've invested in your own confidence, pride, and a healthier future. |
| Food tastes better when you live a smoke-free life! Smoking dulls the taste buds. Savor some delicious food and congratulate yourself for being smoke-free today! |
| Non-smokers have brighter smiles, better breath, and healthier teeth and gums. Be proud of your smoke-free smile! |
| **Encouraging smoking cessation** |
| Smoking causes lung cancer. Try to smoke less tomorrow. |
| Next time you have the urge to smoke, try and resist for 5 minutes. Or skip the cigarette entirely. |
| Value your future. When you quit smoking, you add years to your life. |
| Nobody likes a dirty mouth. After a few days without cigarettes, your smile will be brighter. Quitting smoking will keep your mouth healthy for years to come. |
| Quitting smoking not only helps your lungs heal, but it also improves your night vision. |
| **Supportive of fruit and vegetable consumption** |
| Good job- you ate fruits and vegetables today! Keep up the good work. |
| It's great that you ate fruits and vegetables today. Your balanced diet sets a good example for your children and other loved ones. |
| Great work! A diet rich in fruits and vegetables is key to a healthier life! |
| Keep up the good work and try to eat even more fruits and vegetables tomorrow. |
| **Encouraging increased fruit and vegetable consumption** |
| Fruits and vegetables have essential nutrients, and they make a great snack! |
| A balanced diet is the key to a balanced life! Eat fruits and vegetables to have more energy. |
| Try to eat at least one piece of fruit and one vegetable tomorrow! |
| Start your day the right way-- have some fruit with your breakfast! |
| Try to eat both fruits and vegetables tomorrow! |
| A balanced diet is the key to a balanced life! Eat both fruits and vegetables to have more energy. |
| Fruits and vegetables have essential nutrients, and they make a great snack! |
| A balanced diet needs fruits and vegetables. Eat both, and feel great! |
| **Supportive of not drinking alcohol** |
| You've set a good example for your loved ones by being alcohol-free today. Keep up the good work! |
| Avoiding alcohol gives you more energy. |
| Today, you made the smart choice. Alcohol can cause learning and memory problems. |
| You're building a happier future by avoiding alcohol today- great job! Alcohol abuse can cause depression and anxiety. |
| Living alcohol-free today is a step to a healthier future! Alcohol contributes to heart disease and liver cancer. |
| **Encouraging decreased alcohol consumption** |
| Alcohol can cause liver cancer. Limit your alcohol intake to protect your future health. |
| Alcohol can contribute to poor decisions. Make the smart choice, and make tomorrow an alcohol-free day. |
| Alcohol abuse increases your risk of heart disease. Protect your heart, and stick to water or juice tomorrow. |
| Alcohol isn't just dangerous to your long-term health-- you're more likely to be injured if you've been drinking. Avoid an accident, and make tomorrow an alcohol-free day. |
| Long-term alcohol abuse can cause depression or anxiety. Build a happy future, and live alcohol-free today. |
| **Supportive of condom use** |
| Unprotected sex puts you at risk for HIV. You made the healthy decision by choosing to wear a condom. |
| It's good that you used a condom- be sure to use one next time too! |
| Good! Condoms are the best way to protect yourself against HIV. |
| You made the smart choice by using a condom. Make another smart choice, and get tested for HIV if you don't know your status. |
| **Encouraging increased condom use** |
| Every time you skip the condom, you put yourself and/or your partner at risk for HIV. |
| Always use a condom to reduce your risk of HIV. |
| If you don't know your partner's status, always wear a condom. |
| You should use a condom next time you have sex with a new partner. In the mean time, get tested as soon as possible. |
